# Interobserver variability in quality assessment of magnetic resonance images

**DOI:** 10.1186/s12880-020-00505-z

**Published:** 2020-09-22

**Authors:** Rafal Obuchowicz, Mariusz Oszust, Adam Piorkowski

**Affiliations:** 1 Department of Diagnostic Imaging, Jagiellonian University Medical College, Kopernika Street 19, Cracow, 31-501 Poland; 2 Department of Computer and Control Engineering, Rzeszow University of Technology, Wincentego Pola 2, Rzeszow, 35-959 Poland; 3 Department of Biocybernetics and Biomedical Engineering, AGH University of Science and Technology, Mickiewicza 30, Cracow, 30-059 Poland

**Keywords:** Radiologists, Quality perception, Fleiss’ kappa, Decision process

## Abstract

**Background:**

The perceptual quality of magnetic resonance (MR) images influences diagnosis and may compromise the treatment. The purpose of this study was to evaluate how the image quality changes influence the interobserver variability of their assessment.

**Methods:**

For the variability evaluation, a dataset containing distorted MRI images was prepared and then assessed by 31 experienced medical professionals (radiologists). Differences between observers were analyzed using the Fleiss’ kappa. However, since the kappa evaluates the agreement among radiologists taking into account aggregated decisions, a typically employed criterion of the image quality assessment (IQA) performance was used to provide a more thorough analysis. The IQA performance of radiologists was evaluated by comparing the Spearman correlation coefficients, *ρ*, between individual scores with the mean opinion scores (MOS) composed of the subjective opinions of the remaining professionals.

**Results:**

The experiments show that there is a significant agreement among radiologists (*κ*=0.12; 95% confidence interval [CI]: 0.118, 0.121; *P*<0.001) on the quality of the assessed images. The resulted *κ* is strongly affected by the subjectivity of the assigned scores, separately presenting close scores. Therefore, the *ρ* was used to identify poor performance cases and to confirm the consistency of the majority of collected scores (*ρ*_*mean*_ = 0.5706). The results for interns (*ρ*_*mean*_ = 0.6868) supports the finding that the quality assessment of MR images can be successfully taught.

**Conclusions:**

The agreement observed among radiologists from different imaging centers confirms the subjectivity of the perception of MR images. It was shown that the image content and severity of distortions affect the IQA. Furthermore, the study highlights the importance of the psychosomatic condition of the observers and their attitude.

## Background

The perception of pathologies in the displayed medical resonance (MR) images is often subjective and thus may lead to false-negative errors [22, 24]. Therefore, many clinical studies have been carried out to evaluate the radiological expertise as a part of clinical decision making [25, 32]. Consequently, factors which influence the perception became a matter of scientific discussion, resulting in the foundation of the Medical Imaging Perception Society (MIPS). The society encourages and promotes medical image perception research and education. Such research involves an investigation of physical, social, and behavioral aspects which affect decision-making performance of imaging specialists. Hence, image-dependent and independent factors which strongly influence the perception were identified [11, 38]. They are associated with image creation and processing [20]. A consensus was reached that the best possible resolution and contrast should be ensured to provide an opportunity to recognize anatomical and pathological structures [15, 35]. However, such conditions cannot be met in practice. Therefore, well-defined matrices used in the daily calibration of diagnostic displays are often used to address the quality of displayed image content [31, 34]. To facilitate image interpretation and improve diagnostic performance, display hardware, viewing software, and reading environment are provided in a radiology reading room [17, 41, 44].

Also, a lot of effort was put to define image-independent factors, which are semantic in nature and related to the psychosomatic and sociological aspects of the observed images [5, 21]. They also affect the performance of the cognitive tasks in presence of changes in images [4].

Since distortions are perceived by radiologists it is worth examining the degree of their agreement on the quality of assessed images and determine whether radiologists similarly perceive the quality. To the best knowledge of the authors, the interobserver variability regarding the quality assessment of MR images has not been addressed in the literature. In the existing studies, the discussion mostly covers decisions involving the risk of malignancy based on other than MR imaging methods. For example, in recent works of Pang et al. [30] and Buda et al. [3], the presence of malignancy in ultrasound images and subsequent recommendations were considered. A more developed study presented by Williams et al. [43] involved a subjective assessment of computed tomography coronary angiogram images. In that work, noisy images were used to determine the agreement among radiologists on the diagnosis of angina pectoris due to coronary heart disease for stenosis severity. Sweeney et al. [39], reviews mammographic positioning image quality criteria being the results of years of discussion on the influence of image quality on the detection of breast cancer. Such criteria have been established taking into account observer variability. Performance of radiologists in the identification of cancer cases in mammography images was studied by Rafferty et al. [33].

This study aims at the assessment of a representative group of radiologists in the quality evaluation of MR images. The considered images contain authentic distortions (i.e., they were not artificially introduced) and allow investigating the interobserver agreement among clinicians. The scores for images are also used to determine the individual performance of a clinician using the Spearman rank correlation coefficient, *ρ*. The *ρ* is typically employed to evaluate automatic methods for image quality assessment [37, 41]. This study gives important insight on the variance of the perception of image characteristics in the presence of noise of the group of experienced professionals.

## Methods

### Data collection

The study was performed on a group of 31 radiologists with experience in diagnostic images reading. All medical professionals completed at least 6 years of residency. They are used to work on 1.5T MRI scanners. The study took place in a controlled environment, inside of a lecture room with a limited luminance not interfering with images displayed on monitors. For displaying purposes, Eizo monitors (RadiForce 250) connected to PC computers equipped with dedicated graphics processors (Eizo Quadro) were used. Each observer was equipped with a diagnostic unit and assessed 35 cases (70 images) without interference from other radiologists using grades 1, 2, 3, 4, and 5 which correspond to ’bad,’ ’poor,’ ’fair,’ ’good,’ and ’excellent’ image quality, respectively [14, 40, 42]. The scale of the grades is accepted by the Video Quality Experts Group [40] and is widely used in image quality assessment research [14, 42]. In the presented study, at the beginning of the experiment, two images of the best and worst quality were shown and the grading system was explained. The images were presented simultaneously on all monitors for one minute. Each case consisted of two images of a body structure differing in quality (the double stimulus approach [42]). The participants wrote scores on paper forms to ensure the anonymity of the answers. Then, scores were averaged to obtain the mean opinion score (MOS). The following structures were displayed in different planes: the lumbar and cervical spine (14 images), knee (14), shoulder (16), wrist (6), hip (4), pelvis (4), elbow (2), ankle (2), and brain (8).

The study protocol was designed according to the guidelines of the Declaration of Helsinki and the Good Clinical Practice Declaration Statement. Special care was taken regarding personal data safety, where all the images were anonymized before processing. Written acceptance for conducting the study was obtained from the Ethics Committee of Jagiellonian University (no. 1072.6120.15.2017). Data of 51 patients, 26 men and 25 women, in the age group of 27-41 years, were enrolled in the study. The criteria of negative selection were the image artifacts influencing the image analysis. T2-weighted sagittal sequences of selected body parts were analyzed. To routinely conduct MR studies aiming at decreasing image quality, shortened sequences were made using parallel imaging I PAT software (Siemens). The functionality was implemented using GeneRalized Autocalibrating Partially Parallel Acquisitions (GRAPPA) which resulted in 1.5 min added to the initial exam on the average. Specifically, the GRAPPA 3 was used in which 25% of the echoes were acquired with 60% signal reduction [10]. As a result of the reduced amount of the input data, reconstructed images of the tissue were degraded to lower quality.

The proposed collection was set to represent images of different fields. This is important since the perception of some of them may be different due to the specialization of radiologists in the group (e.g., neuroradiology, gastrointestinal radiology, musculoskeletal radiology, pediatric radiology). It was assumed that the images of the head and spine are more familiar to most participants than those of the remaining parts of the body. Therefore, images of the knee, foot, or wrist were added to the dataset. This may allow determining whether the familiarity with images influences the subjective perception of their quality.

The same protocol was used to collect subjective scores of three interns. The interns were only instructed on the grading scale without any examples of degraded images. Then, the scores of interns were used for the estimation of their performance, while the scores of experienced radiologists were averaged to obtain the MOS characterizing the images in the dataset.

**Fig. 1:**
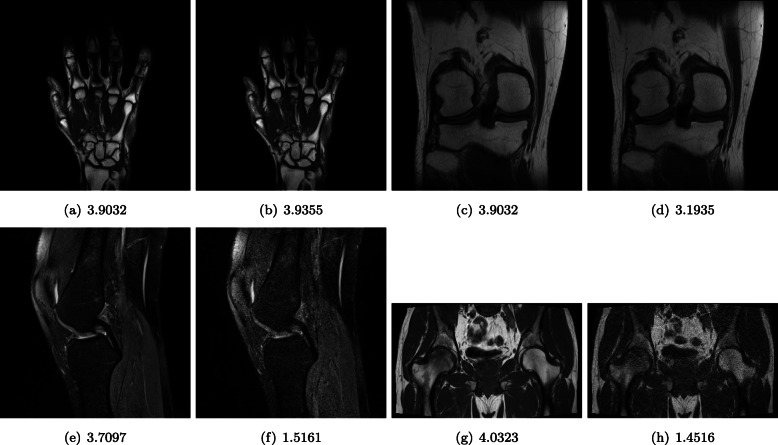
T2-weighted images and their mean opinion scores. The images of the wrist and knee are of the same quality (cf. (**a**) and (**c**)), despite differences in the structure of the tissue and bones. The appearance of structures was perceived worse for the degraded knee (**e**) than shown in (**d**) due to the plane of the acquisition. Severely degraded images of different body parts were assessed similarly (**f**,**h**)

Exemplary image pairs of different body parts and their scores are presented in Fig. 1. It is worth noticing that the scores reflect a subjective perception of noise and its influence on the displayed body part, i.e., while images of a better quality are similarly scored, the scores of their degraded counterparts are different.

### Statistical analysis

Statistical analysis was performed using Matlab [26]. The interobserver variability was assessed using the *κ* statistic. A Fleiss’ *κ* [13] is related to the Cohen’s *κ* statistic. However, it was used since it measures the consistency of the ratings obtained in tests with more than two observers. The *κ* of less than 0 indicated poor agreement, 0.01-0.2 slight agreement, 0.21-0.4 fair agreement, 0.41-0.6 moderate agreement, 0.61-0.8 substantial agreement, and 0.81-1 almost perfect agreement. The test statistics were approximated by a normal distribution to calculate the *p*-value and the 95% confidence interval (CI). Also, since the image quality assessment is considered and the kappa cannot provide a detailed analysis of the individual performance due to the employed aggregation of radiologists’ decisions, the Spearman correlation coefficient, *ρ*, typically used in the IQA field [29, 36, 42], was employed. Subjective scores of a radiologist were compared with the mean opinion score (MOS) calculated as mean scores of the remaining observers to estimate the individual performance.

## Results

**Fig. 2:**
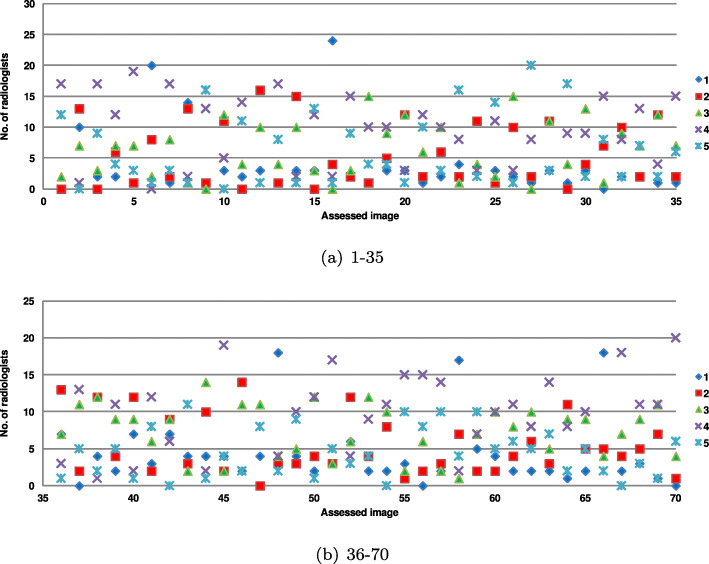
Agreement among radiologists on the image quality expressed by the grades assigned to the images. Images were graded from 1 to 5 by 31 medical professionals

For the entire dataset, the radiologists achieved a *κ* of 0.12 (95% CI: 0.118, 0.121; *P*<0.001), which indicates a slight, but not accidental, agreement. The agreement can also be seen in Fig. 2 in which the number of radiologists assigning a given grade for an image is reported. Only 19 images were assigned the same grade by more than half radiologists. Interestingly, 11 images were assigned two close grades by the same number of radiologists. For example, the image shown in Fig. 1d was assigned ‘3’ and ‘4’ by 10 specialists (cf. no. 22 in Fig. 2). There are also some images with two close scores (e.g., Fig. 1g, image no. 15 in Fig. 2, was graded ‘4’ and ‘5’ by 12 and 13 radiologists, respectively).

**Fig. 3:**
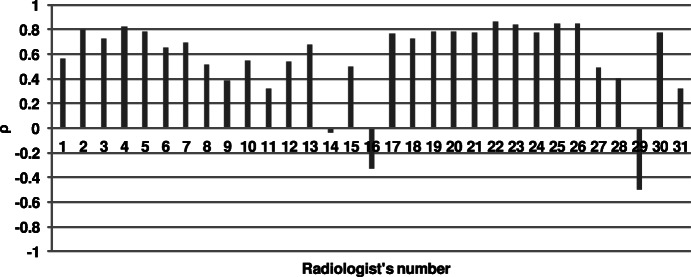
Evaluation of radiologists’ IQA performance in terms of Spearman correlation coefficient (*ρ*)

To evaluate decisions of radiologists’ from image quality perspective, they were correlated with average decisions of the other professionals (Fig. 3). Such an examination takes into account close differences between scores for images instead of aggregated totals used for the calculation of the *κ*. Consequently, this widely-accepted method for the evaluation of automatic IQA measures was used to provide a more detailed analysis of radiologists’ performance. The obtained average, maximum, minimum, and standard deviation of the *ρ* are 0.5706, 0.8615, -0.4988, 0.3331, respectively. The correlation coefficients reveal a large variability among them, due to weaker or unexpected performances of several specialists. Specifically, the performance of three radiologists affected the results. The negative correlations for 16th and 29th radiologists may evidence their lack of understanding of the used grading system. However, the resulted negative correlations show that they can evaluate the images. More important is the result for the 14th radiologist who seems to assessed images disregarding their quality.

**Fig. 4:**
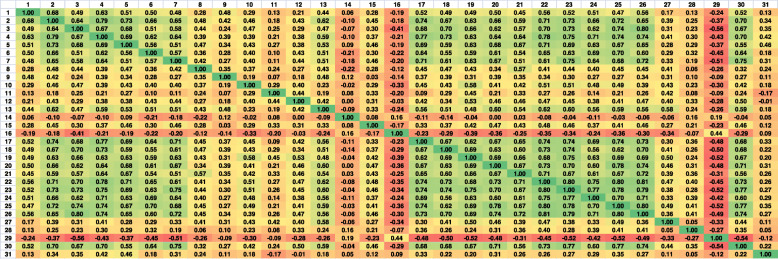
Spearman correlation coefficients between scores of radiologists

To determine the individual agreements between radiologists, in terms of the IQA, the *ρ* in pairs was calculated (Fig. 4). The obtained values reflect moderate to the strong correlation of scores in pairs of medical professionals. The lack of agreement of the 14th radiologists with other specialists is also highlighted in this experiment. The findings confirm the previously reported individual results and reveal that most observers’ opinions are moderately (to strongly) correlated with those of other professionals.

Once the performance of experienced radiologists was evaluated, the IQA performance of three interns who assessed the MR images for the first time was examined. The interns were only instructed on the grading scale. The following results, in terms of the *ρ*, were obtained: 0.7450, 0.6733, and 0.6419. They confirm that even an inexperienced observer can differentiate the images based on their quality.

**Fig. 5:**
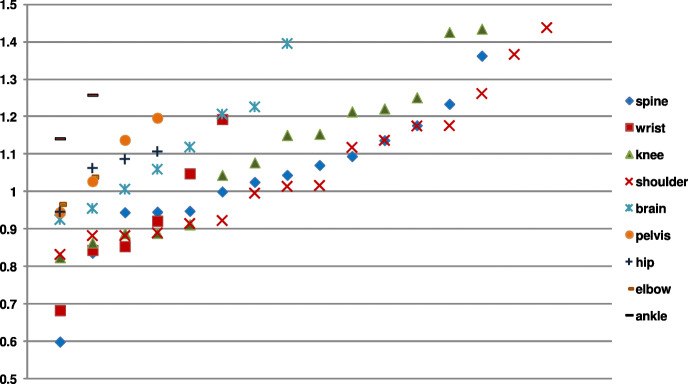
Sorted standard deviations of scores for images of body parts

Since the dataset contains images of different body parts, the agreements of the radiologists expressed by the *κ* as well as the *ρ* were reported (Table 1). In all experiments, the obtained agreements are slight (*κ*∈(0;0.2]) and significant (*P*<0.001). For parts of the body with two images (i.e., the ankle and elbow), some radiologists assigned them the same grades, preventing the calculation of the *ρ*. In such cases, the remaining values were averaged. However, mean values for images of separate body parts are close to those obtained for the entire dataset. The reported high maximum values show that the observers’ opinions on the image quality were consistent, despite the opposite quality perception of several of them. The last two rows of the table show results for groups of images. Here, frequently examined parts of the body (i.e., head and spine) were considered jointly. Also, the scores for the remaining images were used in calculations. As reported, the performance of radiologists assessing these two groups of images does not vary much and the quality of images was scored similarly even for rarely considered parts of the body in daily work. This is supported by the standard deviation of scores for images shown in Fig. 5 which reveal large differences of scores for some images of the knee and shoulder, closely followed by scores for images of the brain and spine.

**Table 1:** Interobserver variability in the IQA of MR images of different body parts

	*κ*	Confidence interval		*p*-value	*ρ*_*mean*_	*ρ*_*max*_	*ρ*_*min*_	*ρ*_*std*_
All images	0.120	0.118	0.121	<0.001	0.5706	0.8615	-0.4988	0.3331
Spine	0.106	0.103	0.110		0.5944	0.9044	-0.3188	0.3156
Knee	0.114	0.111	0.117		0.5527	0.9108	-0.5202	0.4321
Shoulder	0.128	0.125	0.131		0.5839	0.9304	-0.6490	0.4009
Wrist	0.105	0.100	0.110		0.4581	0.9258	-0.7356	0.3982
Hip	0.048	0.042	0.055		0.4136	0.9487	-0.9487	0.6648
Elbow	0.079	0.071	0.088		0.9231	1.0000	-1.0000	0.3922
Ankle	0.089	0.081	0.098		0.6000	1.0000	-1.0000	0.8137
Brain	0.084	0.080	0.088		0.4966	0.9698	-0.8230	0.5786
Spine ∪ Brain	0.102	0.100	0.105		0.5774	0.9052	-0.4421	0.3606
All - (Spine ∪ Brain)	0.118	0.117	0.120		0.5671	0.8951	-0.4824	0.3286

## Discussion

Perception of the image is fundamental for diagnostic imaging professionals. Radiological training is directed toward critical analysis of the possible abnormalities present in the image. Therefore, diagnostic image assessment relies on the methodological analysis of the displayed content representing human anatomy. However, to perform the analysis a plethora of possible pathological changes as well as anatomical variants should be taken into account. Since the quality perception of radiological images and its relationship with the diagnostic image assessment is seldom addressed in the literature, in this paper, the agreement among professionals on the quality of MR images is studied. The aim of the study was to determine whether the decisions on the quality of a group of radiologists are in agreement. Consequently, this may indicate that the professionals similarly perceive MR images acknowledging the severity of the observed distortions.

This study showed that decisions on the quality are in a slight agreement (*κ* = 0.12; 95% CI: 0.118, 0.121; P <0.001). However, due to the subjectivity of the quality assessment and range of scores assigned to the images (1-5), such a result is not surprising. Therefore, the obtained scores were further analyzed using the *ρ*, which is typically employed for the evaluation of the ability of the automatic image quality assessment techniques to mimic human perception and provide objective scores for images. The radiologists were separately evaluated, and the reported *ρ*=0.5706 allows concluding that they similarly perceive distortions in MR images.

**Fig. 6:**
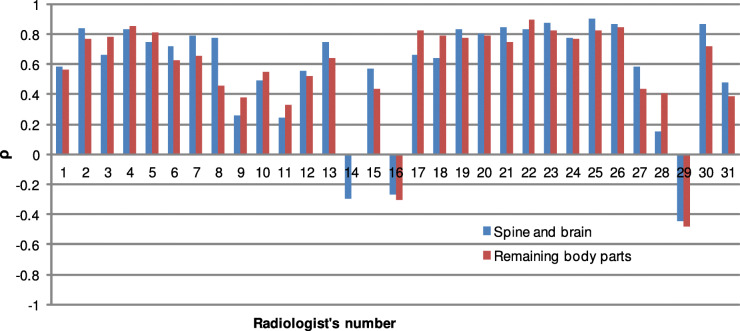
Comparison of image quality assessment performances of radiologists evaluating MR images of familiar (i.e., brain and spine) and unfamiliar body parts. The performances are similar in both cases as the mean *ρ* for brain and spine images is 0.5774, while for the remaining images 0.5671

Furthermore, more detailed tests were also carried out in which familiar images were used jointly. Since the group of radiologists was far more familiar with neuroradiology than with the musculoskeletal radiology, the influence of work experience of professionals on the perceived quality could be examined. As reported in Table 1 and Fig. 5, the correlation between radiologists’ scores for neuroradiology images represented by the subset of spine and brain images were similar to correlations obtained for the subset of images of different joints representing musculoskeletal radiology. To support these observations, Fig. 6 contains the *ρ* values for radiologists in both cases.

**Fig. 7:**
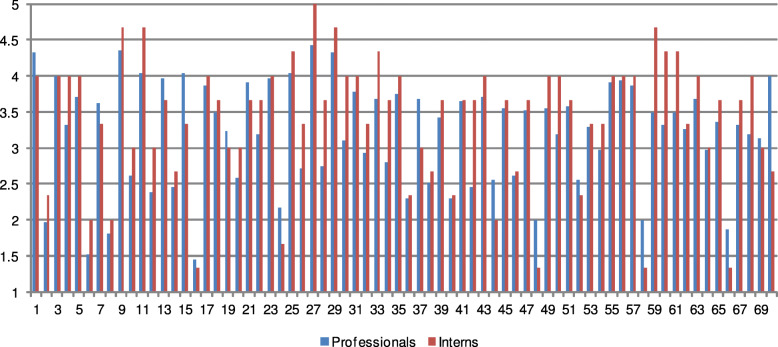
Mean opinion scores of professionals and interns for images

Also, the experiments which involved interns revealed no significant influence of professional background in the quality assessment. Their average *ρ* is 0.6867 and is higher than the average result for experienced radiologists (*ρ*=0.5706), demonstrating that the correct assessment can be performed even by an inexperienced observer. This can be also seen in Fig. 7, in which mean opinion scores for images are shown separately for professionals and interns. This is in contradiction to the work of Miao et al. [28] who assumed that radiologists have an advantage in the critical analysis of the images in which quality differences are present. However, such a claim was corrected in their further study [27]. In contrary to both studies, in which only up to two radiologists took part, the findings presented in this paper are based on decisions of a much larger group of medical professionals.

**Fig. 8:**
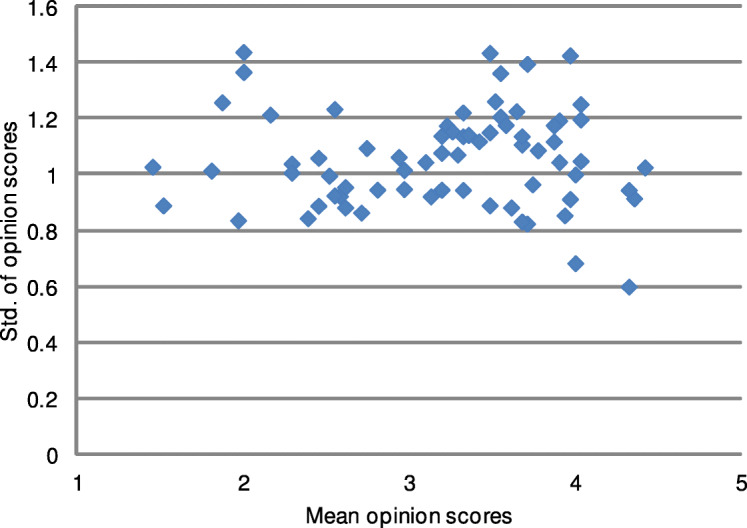
Dispersion of opinion scores for images assessed by professionals. The differences between scores can be seen for images of different quality, indicating that the perception of displayed body parts and personal preferences of observers also took part in the image assessment

**Fig. 9:**
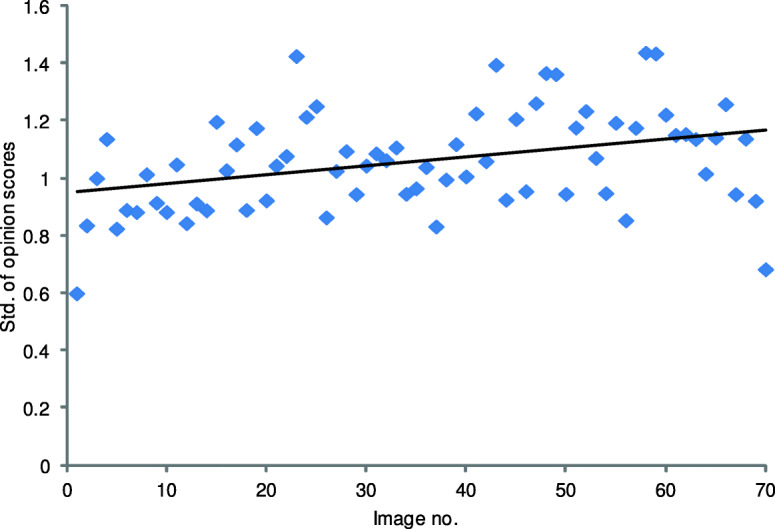
Dispersion of opinion scores for images during the test

Furthermore, this study reveals that the content of images strongly affects their perceived quality. As can be seen in Fig. 8, dispersion of scores for images vary much for images of medium quality. The images of the worst quality were unanimously assessed by the group since they contain visible noise or distorted contours of the displayed shapes. Consequently, images of the best quality are also characterized by a relatively small standard deviation of the scores. This indicates that the decisions of radiologists are consistent. Interestingly, as pointed out by Daly [8], a group of imaging professionals trained for the recognition of changes in the grayscale scene may be able to successfully use images of a low quality. To further investigate the dispersion of scores during the experiment, Fig. 9 shows their deviations for consecutive images. As revealed by the trend line, the standard deviation of scores slightly increases over time. It can be assumed that a longer duration of the test would negatively affect the performance of the group of radiologists. However, the observed trend is not strong since the experiment was fairly short to reduce the fatigue of the participants.

In the group of examined professionals, a moderate linear relationship between opinion scores was reported. This confirms the consistency of the majority of collected subjective opinions and highlights the interobserver agreement on the image quality. However, the scores of a few professionals are negatively correlated with the rest of the group which suggests that they did not use the established image grading system and assigned scores in the reversed scale. The correlation coefficients indicate that they were aware of the differences in the distortion severity of the assessed images as the rest of the group. The usage of the reversed scale may also show the importance of the overall attitude and psychosomatic status in the work with images [25]. In contrary to other medical professions, in radiology, there is a blind (one-way) interaction with displayed content which demands self-control and criticism.

The presented study was carried out on a representative group of radiologists and focused on the recognition of differences in the quality of MR images. The best of our knowledge such an approach is presented for the first time. Specifically, studies regarding quality in the diagnostic imaging proposed to date are directed towards the analysis of the influence of the image quality distortions on the perception of images [43]. Also, Sweeney et al. [39] and Rafferty et al. [33] presented findings on the influence of image quality on the perception of the pathology. In that work, images were artificially distorted using blur or noise. Influence of the different algorithms used for the raw image post-processing techniques on the image quality and their final perception by radiologists can be found in the literature [1, 2, 7]. Also, the analysis of the influence of image acquisition on the radiological perception of different pathologies in an various radiological modalities is often considered [6, 9, 12, 16, 18, 19, 23, 45]. However, these works lack an investigation of the level of the agreement among professionals assessing the quality of MR images.

The size of the group of radiologists as well as the number and diversity of the assessed images can be seen as the limitations of this study. However, to the best of our knowledge, this is the first time a large number of radiologists is involved in the assessment of the quality of images. Also, the choice of the images for the study is not accidental as they show typically examined body parts and parts with which most of the professionals are not familiar to study how their experience affects the perceived quality. Furthermore, the radiologists taking part in the study were familiar with the output of the employed 1.5T MRI scans as they work on machines of this filed strength. Consequently, assuming that the assessment of 3T MRI scans could be difficult for the professionals used to 1.5T images, the experimental setup applied in this study considers only 1.5T MRI scans to provide conditions that did not distracted participants.

## Conclusions

This paper discusses the interobserver variability in the assessment of MR images. The variability was evaluated using opinion scores of the group of experienced medical professionals and interns, reflecting their assessment of a dataset of authentically distorted MR images. The observed agreement in the group of radiologists from different imaging centers confirmed that the perception of the image quality is subjective and depends on the meaning of the displayed shapes, contours, and grayscale differences responsible for the essential cognition of the image. It was determined that the quality assessment is only partially influenced by the distortion severity and is correlated neither with the knowledge on the anatomical representation of the structures nor the experiences on image perception. However, it was influenced by the psychosomatic condition and attitude of the observers.

Future work would be focused on an investigation of a group of professionals assessing medical images from different radiological modalities or an investigation of a degree of agreement among repeated examination of images in a form of intraobserver tests.

## Data Availability

The dataset supporting the conclusions of this article is available in the mriqdataset.7z repository: http://home.agh.edu.pl/pioro/mriqdata/.

## Abbreviations

CI: Confidence interval
IQA: Image quality assessment
MOS: Mean opinion scores
MRI: Magnetic resonance imaging
